# A Dried Yeast Fermentate Prevents and Reduces Inflammation in Two Separate Experimental Immune Models

**DOI:** 10.1155/2012/973041

**Published:** 2012-04-05

**Authors:** Malkanthi Evans, Stuart Reeves, Larry E. Robinson

**Affiliations:** ^1^KGK Synergize Inc., 255 Queens Avenue, Suite 1440, London, ON, Canada N6A 5R8; ^2^Embria Health Sciences, 2105 Creekview Drive, Ankeny, IA 20021, USA

## Abstract

Diverse and significant benefits against cold/flu symptoms and seasonal allergies have been observed with a dried fermentate (DF) derived from *Saccharomyces cerevisiae* (EpiCor) in multiple published randomized trials. To determine if DF may influence other immune conditions, two separate animal studies were conducted. Study 1 examined the ability of DF to prevent or reduce inflammation when given orally for 14 days to rats prior to receiving 1% carrageenan (localized inflammation model). DF significantly (*P* < 0.05) reduced swelling at all time points (1, 2, 3, 6, 12, and 24 hours) versus the control. Edema severity and PGE2 levels were reduced by approximately 50% and 25% (*P* < 0.05), respectively. Study 2 examined the ability of DF to treat established inflammation induced by type-2 collagen in mice over 4 weeks (autoimmune arthritis model). Significantly reduced arthritis scores, antibody response to type-2 collagen, and interferon-gamma levels were observed compared to controls (all parameters *P* < 0.05). DF favorably impacts multiple acute and potentially chronic immunologic inflammatory control mechanisms and should be further tested in clinical trials.

## 1. Introduction

Concerns abound over the dearth of basic science and clinical data with some prescription cold, allergy, and other immune-based agents, which is highlighted by recent concerns from the Food and Drug Administration (FDA) and the subsequent removal of numerous products from the market [1]. Issues and ambiguities with over-the-counter (OTC) products appear just as problematic and need to be monitored [2, 3]. Clinical and basic science research on the efficacy, safety, quality control, and mechanisms of action are needed for any commercially available product.

The dried fermentate product (DF) derived from *Saccharomyces cerevisiae* (EpiCor, Ankeny, IA) has now been studied in five randomized, double-blind, placebo-controlled clinical trials with over 300 adult participants [4–8]. The data from these published trials suggest multiple mechanisms of action compared to conventional and nonconventional products. DF has demonstrated the potential to favorably modulate immune responses without excessive suppression or stimulation of overall immune activity. This conclusion is based on multiple clinical observations in trials, which also consistently demonstrated the safety of this product (including minimal adverse events which were similar to placebo). DF demonstrated significant clinical benefits in reducing the incidence and duration of cold- and flu-like symptoms during fall and winter months, regardless of influenza vaccination status [4, 5]. The daily consumption of DF significantly increased salivary IgA levels [6, 7] while at the same time reducing pollen-associated allergy symptoms such as nasal congestion during spring and summer months [6].

An intervention that can impact immune activity in some capacity throughout the year might also be providing several other subtle physiological benefits. It remains a priority to further elucidate these mechanisms of action based on both laboratory research and clinical trial outcomes and provide further clarity into the strengths and limitations of DF. Thus, two well-established and accepted animal models of immune surveillance were utilized that focus on anti-inflammatory effects of an intervention [9, 10]. The studies were to determine if DF could either prevent (study 1) [9] or treat (study 2) [10] an induced inflammatory response.

## 2. Materials and Methods

### 2.1. Animal Care and Housing

All animal procedures for the study were approved by the Animal Care and Use Committee of the University Council on Animal Care, University of Western Ontario, London, Ontario, Canada and were performed according to guidelines of the Canadian Council on Animal Care. All animal procedures were performed with the strictest adherence to these guidelines concerning humane handling and treatment throughout the study and included such items as ideal light and dark cycles (12 hours), temperature (18–26 Celsius), relative humidity (30–70%), and availability of food and water (*ad libitum*). Both the rats and mice were randomized so that the variation in weight in each group did not exceed two standard deviations of the mean body weight.

### 2.2. DF Fermentate Preparation

EpiCor, which in this paper is referred to as “DF,” was provided by the manufacturer of the product (Embria Health Sciences, Ankeny, IA) as a powder comprised of the heat-inactivated yeast *Saccharomyces cerevisiae* and the fermentation broth milieu that was utilized for growth. DF was prepared weekly in a solution of 75% glycerol and 25% deionized water to a final concentration of 1 mg/mL for oral gavage delivery. The volume administered was adjusted to the mean body weight of each animal group.

### 2.3. Study 1 Inflammation Prevention/Reduction Model

A total of 20 Sprague-Dawley male rats (Charles River, Saint Constant, QC), approximately 6–8 weeks of age at baseline, were housed individually and acclimatized for one week prior to the initiation of the study. After the acclimatization period, the animals were weighed and randomly assigned based on body weight using a computer-generated sequence ensuring mean body weights were not statistically significant between groups. Animals were assigned to DF treatment (*n* = 10) or control (*n* = 10) and were utilized for the localized inflammatory prevention model. Rats ingested DF (7 mg/kg body weight) or control by oral gavage for 14 days. After the treatment period (day 15), the left-hind footpads were injected with 50 *μ*L of 1% carrageenan (saline diluted), and the right-hind footpads with 50 *μ*L of saline. Paw volume changes were monitored with a digital plethysmometer (IITC LifeScience, Woodlands, CA) at baseline, 1, 2, 3, 6, 12, and 24 hours (end of the study period) after the injection. Approximately, 100 *μ*L of blood was collected from the saphenous vein of each rat prior to injection. The effects of DF on prostaglandin E2 (PGE2) and nerve growth factor (NGF) concentrations in paw tissue in response to carrageenan and saline injection were also measured. Rats were sacrificed and paws removed and degloved. Tissue was transferred into 50 volumes of ice-cold homogenization buffer and homogenized by repeated cycles of 20-second ultrasonic disruption followed by 10-second recovery. These cycles were continued until a homogenous slurry was produced using an ultrasonic cell disruptor (Misonix, Farmingdale, NY). The homogenization buffer contained 100 mM Tris/HCl, pH 7, 1 M NaCl, 4 mM EDTA.Na_2_, 2% Triton X-100, 2% bovine serum albumin, 0.1% sodium azide, 5 *μ*g/mL aprotinin, 0.5 *μ*g/mL antipain, 157 *μ*g/mL benzamide, 0.1 *μ*g/mL pepstatin A, and 17 *μ*g/mL phenylmethyl-sulfonyl fluoride. The homogenates were centrifuged at 14,000× g for 30 minutes. The supernatants were transferred to microfuge tubes and stored between −70 and −90 Celsius until analysis for prostaglandin E2 (PGE2) and nerve growth factor (NGF) by enzyme-linked immunosorbent assay (ELISA).

Tissue extracts and serum from the 6 time points from each rat were analyzed for the presence of PGE2 using a Prostaglandin E Metabolite kit (Cayman Chemicals, Cat. 514531, Ann Arbor, MI). The assay was performed according to manufacturer's protocol. Tissue extracts were diluted 1 : 640, and the serum samples were diluted 1 : 200 for the 0–12-hour samples or 1 : 80 for 24-hour serum samples. PGE2 levels were expressed relative to wet tissue weight. Tissue extracts were analyzed for the presence of NGF from each rat using an NGF Emax ImmunoAssay kit (Promega, Cat. G7630, Madison, WI). The assay was performed according to manufacturer's protocol. Tissue extracts were utilized undiluted in the assay, and NGF levels were expressed relative to wet tissue weight.

### 2.4. Study 2 Inflammation Treatment Model

A total of 20 DBA/1J male mice (The Jackson Laboratory, Bar Harbor, ME), approximately 8–10 weeks of age at baseline, were group housed and acclimatized for one week prior to the initiation of the study. After the acclimatization period, the animals were weighed and randomly assigned based on body weight using a computer-generated sequence ensuring mean body weights were not statistically significant between groups. Animals were assigned to DF treatment (*n* = 10) or control (*n* = 10) and were utilized for the inflammation treatment model. Mice were injected with type II collagen (bovine collagen type II, Sigma catalog no. C1188, Oakville, ON) at the base of the tail, and a booster injection (intraperitoneal) of collagen was delivered 3 weeks after the initial immunization. Upon appearance of arthritis (in greater than 80%), mice were orally gavaged with either DF (7 mg/kg body weight in 75% glycerol) or control (75% glycerol) daily for 4 weeks (end of the study period). Arthritis was evaluated based on daily examination of erythema and swelling (edema). A 0–4 scoring system was utilized and monitored daily with 0 as no evidence of erythema and swelling and 4 as the maximum amount comprising the ankle, foot, and digits. The sum of the clinical scores of the 4 paws yielded a sum score (maximum score of 16 for each mouse). Swelling was monitored daily by measuring ankle (medial/lateral) and tarsal (dorsal/ventral) thickness with digital calipers. Blood was collected by saphenous venipuncture at the onset of arthritis for the measurement of anticollagen type II antibody. Cytokine levels (IFN-*γ*, TNF, and IL-1*β*) were detected in mouse serum using commercial ELISA kits (BD Biosciences; BD OptEIA Set Mouse IFN gamma, Cat. 555138; BD OptEIA Set Mouse TNF, Cat. 555268; BD OptEIA IL-1beta, cat. 559603, Becton Dickenson, Mississauga, ON) and following manufacturer's instructions. ELISA plates were read on a MultiSkan Ascent Plate Reader (Thermo Scientific, Middletown, VA), and data were analyzed using Ascent Software, version 2.6.

### 2.5. Statistical Analysis

Analysis of body weights, arthritic scores, cytokine data, and edema measurements was performed using one-way analysis of variance (ANOVA) followed by Holm-Sidak test. Significance of both tests was determined if *P* < 0.05. Differences of means of NGF, PGE2, and collagen antibody concentrations in serum and tissue samples and differences of means for paw measurements were analyzed using Student's *t*-test.

## 3. Results

**Study 1: Inflammation Prevention/Reduction Model**

**Figure 1:**
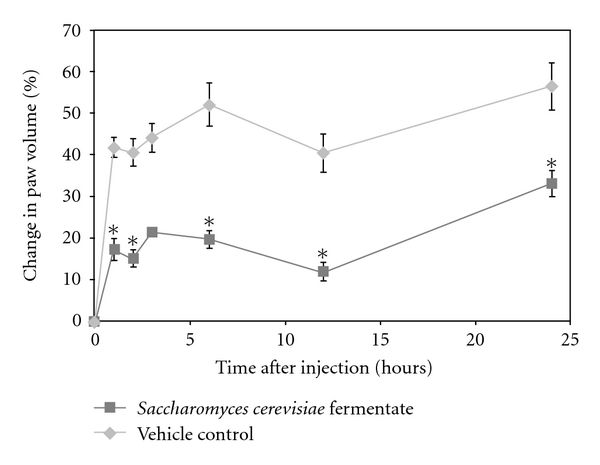
Effects of carrageenan injection on paw volumes in rats pretreated with *Saccharomyces cerevisiae* fermentate (DF) or vehicle. The figure shows group means ± SEM for each time point. Asterisk (*) denotes a significantly (*P* < 0.05) lower percentage change in paw volume for DF-fed rats compared to those on vehicle control.

**Figure 2:**
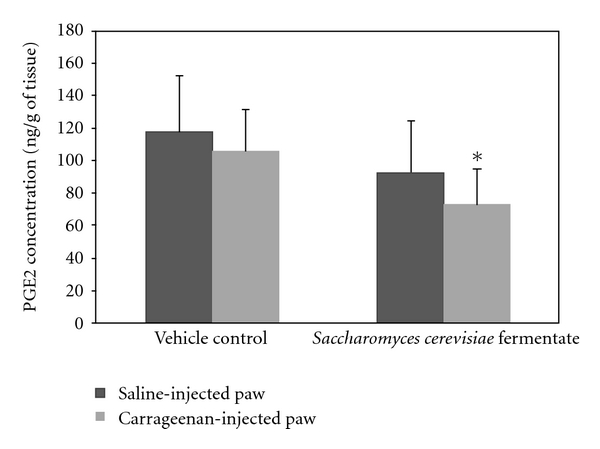
Effects of carrageenan injection on PGE2 concentrations (ng/g paw tissue) for *Saccharomyces cerevisiae* fermentate- (DF-) fed rats compared to vehicle-fed rats. The figure shows group means ± SD. Asterisk (*) denotes a significantly (*P* < 0.05) lower PGE2 concentration in DF-fed rats compared to those on vehicle control.

The impact of a 14-day preventive treatment with DF compared to controls was determined. The percentage changes in paw volumes in the carrageenan, compared to the saline paws, were significantly different (*P* < 0.05), as was expected from this model. The percentage change in paw volume for the carrageenan-injected rats given DF compared to vehicle was significantly less (*P* < 0.05) at all time points (Figure 1). In the case of the vehicle and DF-treated saline-injected rats, it is of interest to note that the inflammatory response was less in the DF-treated rats at all time points, although this was not statistically significant. PGE2 in paw tissue from carrageenan-injected rats was significantly (*P* < 0.05) reduced for DF-fed compared to vehicle-fed animals (Figure 2). Levels of PGE2 were lower in the saline paws of DF-treated rats, but the results were not significantly different. Though there was a decrease in PGE2 concentrations in the carrageenan-injected paws compared to saline-injected paws of vehicle-fed rats, the difference was not significant. The increase in PGE2 concentration in saline-injected paws is expected in vehicle-fed rats and may be attributed to an inflammation resulting from the site of injection. There was also a nonsignificant 22% reduction in NGF levels in the DF group compared to the vehicle group. NGF levels were significantly lower (*P* < 0.05) in the saline compared to the carrageenan-injected paws in the control group demonstrating the impact of this compound on inducing increased levels of tissue NGF. No statistical differences in the weights of the animals were observed during the entire testing period (data not shown).

**Study 2: Inflammation Treatment Model**

**Figure 3:**
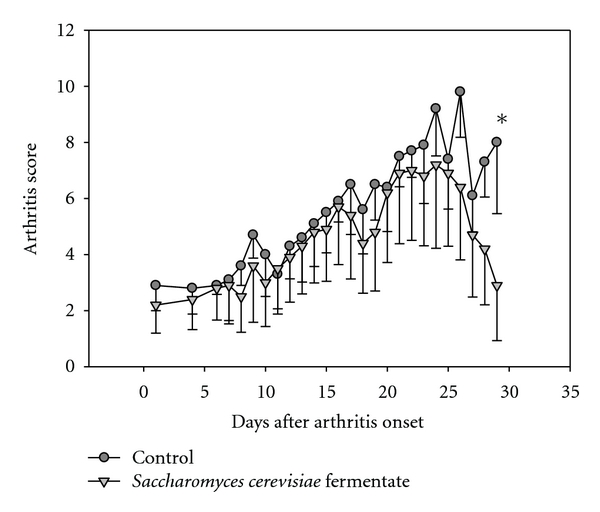
Effect of treatment with *Saccharomyces cerevisiae* fermentate (DF) or vehicle on the progression of the development of arthritis. Treatment with DF decreased the severity of collagen-induced arthritis between days 18 and 29 after the onset of arthritis in >80% of the mice. Day 0 indicates the day of the booster injection. Mice were gavaged daily with treatment or vehicle. The figure shows group means ± SD for each time point. Asterisk (*) denotes a significant (*P* < 0.05) reduction in arthritis scores in DF-gavaged mice compared to vehicle-gavaged mice.

**Figure 4:**
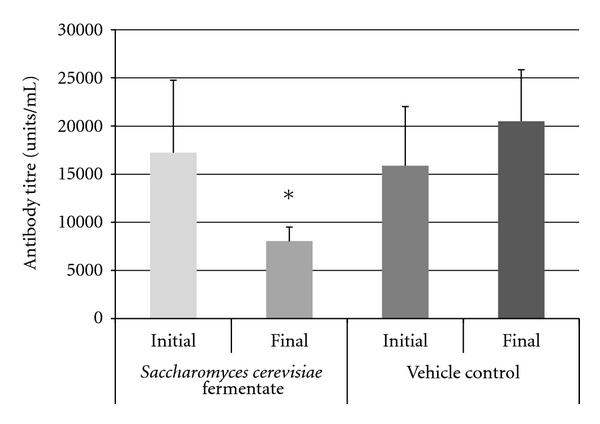
Antitype II collagen antibody titers in arthritic mice treated with *Saccharomyces cerevisiae* fermentate (DF) or with vehicle control. DF treatment blocked increases in antitype II collagen antibody titers. The figure shows group means ± SEM. Asterisk (*) denotes a significant (*P* < 0.05) reduction in antitype II collagen antibody titers in DF-gavaged mice compared to vehicle-gavaged mice.

The impact of treatment with DF compared to controls after arthritis was detected and conducted. The arthritis scores in the DF and control arms were similar until day 17. A reduction in the arthritic scores was observed between days 18 to 29 in the DF-treated mice as compared to the control group. This reduction continued until the study termination, and the scores in the DF mice were significantly (*P* < 0.05) lower than the control mice (Figure 3). There was no difference in antibody levels between DF treated and controls at the onset of arthritis. DF treatment significantly (*P* < 0.05) inhibited the antibody response to type 2 collagen compared to controls by the end of the study (Figure 4).

**Figure 5:**
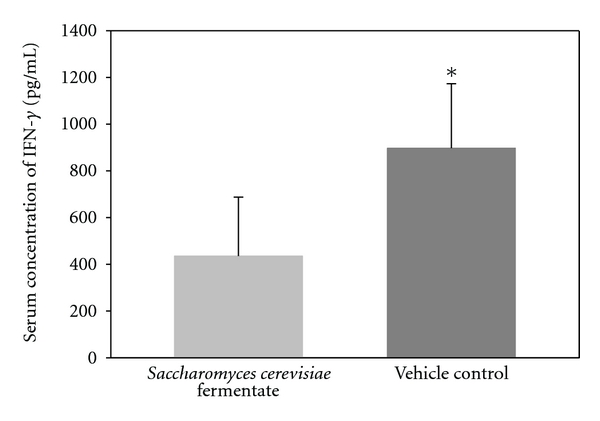
IFN-*γ* levels in arthritic mice treated with *Saccharomyces cerevisiae* fermentate (DF) or with vehicle control. The figure shows group means ± SD. Asterisk (*) denotes a significant (*P* < 0.05) increase in IFN-*γ* levels in mice-gavaged vehicle compared to those gavaged DF.

No detectable concentrations of TNF or IL-1*β* were found in the mice treated with the DF or controls. In contrast, IFN-*γ* levels were significantly (*P* < 0.05) lower in the DF group compared to controls at the end of the study period (Figure 5). No statistical differences in the weights of the animals were observed during the entire testing period.

## 4. Discussion

DF consists of a dried powder of novel yeast fermentate comprised of both the heat-inactivated yeast (*Saccharomyces cerevisiae*) and the fermentation broth in which it was grown. This product contains a high concentration of metabolites and numerous and diverse-free radical scavenger compounds as exemplified by its high antioxidant content as shown in the oxygen radical absorbance capacity (ORAC) assay [11]. DF antioxidants have already demonstrated the ability to gain access to live cells both *in vivo* and *in vitro* thereby providing additional cellular protection [8, 12]. Furthermore, five randomized, double-blind, placebo-controlled trials have demonstrated the efficacy and safety of this dietary supplement and have been published in peer-reviewed journals [4–8]. Several mechanisms of action were suggested from the clinical trials and *in vitro* studies, such as enhanced salivary IgA production, NK cell activation, and increased antioxidant capacity [6–8, 11, 12]. Still, further mechanism of action research was needed to provide a more lucid explanation of some of these observed clinical benefits as well as the potential for utilizing this product for other immune-based conditions. A product that reduces cold- and flu-like symptoms and seasonal allergy symptoms theoretically appears to provide both moderate immune enhancing as well as moderate anti-inflammatory benefits. Studies 1 and 2 in this paper were carried out to provide more insight into possible anti-inflammatory properties of DF *in vivo*.

The induction of paw swelling with carrageenan in study 1 was successful as expected, since this model has become a standard for inflammation studies over time [9, 13]. However, the observation that DF significantly reduced paw swelling in this model at every time point was a consistent positive and unexpected finding, suggesting an acute and durable or potentially chronic response with DF. A greater than 50% reduced swelling response was observed at all but one time point after injection. A major mediator of the localized inflammatory response in this model is the proinflammatory prostaglandin PGE2 [14], which has a role in other medical conditions as well. For example, in autoimmune diseases such as rheumatoid arthritis, PGE2 has a proinflammatory function [15]. The production of both IL-23 and IL-17 is induced by PGE2, and these compounds are implicated in rheumatoid and systemic joint destruction, and perhaps even “wear and tear” localized joint osteoarthritis [16, 17]. This study demonstrated a significant reduction in carrageenan-induced tissue concentrations of PGE2, which now provides some information into how DF may be providing more specific immune benefits. Prostaglandins, including PGE2, are also produced in large amounts during allergen exposure [18], and some conventional medicines reduce PGE2 levels as part of their respective mechanisms of action [19]. A significant reduction in nasal congestion from seasonal allergies found in a past clinical trial utilizing DF can now be partially explained from the favorable impact on PGE2 in this study [6]. It is also of interest that a traditional Chinese medicine (Guizhi-Tang or GZT) utilized to treat the common cold, pyrexia, and influenza is thought to favorably impact several immune pathways including the fairly recent finding of a reduction in PGE2 [20]. This medicine also contains a plethora of diverse compounds [21]. These observations may provide a partial explanation of the favorable effects with DF in significantly reducing the duration and incidence of cold and flu symptoms in clinical trials of vaccinated and nonvaccinated individuals [4, 5].

Increases in NGF are associated with discomfort and pain with inflammatory responses because it impacts mast cells and afferent neurons [9, 22]. The excessive production of NGF found in past studies of allergic rhinitis patients is again of interest [23, 24], because one of our past DF clinical trials provided tangible symptomatic benefits against this condition [6]. Recent research has also suggested that a mechanistic route for a future efficacious pain ameliorating medication may be via an anti-NGF mechanism [25, 26]. NGF is such a profound primary mediator of chronic pain that even vaccine research has been initiated in this area of medicine [27]. The moderate but significant reduction in NGF observed in our study in DF-fed rats with carrageenan-injected paws compared to controls may be another explanation for the immune modulating impact of DF. Yet, it must be kept in mind that NGF also appears to inhibit immune suppressive signaling and promotes temporary steroid receptor insensitivity in some leukocytes [28–30]. In other words, again the interplay of multiple compounds involved in immune activity suggests that modulation rather than excessive reduction or stimulation would explain some of the clinical findings with DF [4–8].

Study 2 utilized the type II collagen-induced inflammatory (arthritis) mouse model that has also been widely used as a standard autoimmunity model [31]. Collagen injection rapidly induces arthritic-like symptoms with associated changes including swelling, erythema, and discomfort. Cellular and humoral arms of the immune response to collagen both contribute to inflammation via disease-exacerbating proinflammatory cytokines and growth factors [32]. In this study, the arthritis scores in the DF and control arms were similar until day 17. However, by the end of the study period, the scores were significantly lower in the DF mice compared to the controls. These tangible results were potentially further strengthened with the observation of a significant reduction in the anticollagen type 2 antibody titers and IFN-*γ* concentrations, which also suggests a potential modulation of Th1 subsets of T helper cells [33].

IFN-*γ* is one of the primary endogenous mediators of inflammation and immunity [34, 35]. IFN-*γ* has a diverse role including macrophage activation, tissue remodeling, host defense, and even enhancement of autoimmunity. Classic autoimmune conditions such as inflammatory bowel disease, lupus, multiple sclerosis, psoriasis, and rheumatoid arthritis display large concentrations of activated macrophages at inflammatory sites. These macrophages are believed to enhance the production of cytokines such as IL-6 and TNF. Hence, IFN-*γ* has been considered an autoimmune disease promoting or proinflammatory cytokine, as also suggested from the collagen-induced arthritis model. However, the complexity and diversity of the immune system and DF for that matter is greater than previously appreciated. IFN-*γ* is now thought to have pleiotropic effects and thus can have both promoting and suppressive roles in autoimmunity [36]. For example, in a previous study [8], feeding DF to human subjects caused a rapid but transient increase in IFN-*γ*. Regardless, the moderate but significant ability of DF to suppress IFN-*γ* in study 2 appears to suggest one favorable anti-inflammatory pathway, and perhaps any further suppression would not necessarily suggest an enhanced positive outcome without some negative consequences. This supports a selective action of DF, dependent on the health of the mammal and the timing of the sampling. This again can be related to clinical studies whereby DF was clinically beneficial but without adverse events compared to a placebo.

The limitations of these 2 studies were primarily the one-time end-of-study biomarker measurements. Blood sampling in the early to midphase of disease development would be prudent; however, adhering to strict ethical animal guidelines allows for a limited amount of blood to be drawn from each mouse or rat, so only the end-of-study points were feasible. TNF and IL-1*β* did not change appreciably in DF treated or control, which would suggest that these are not suitable markers for ongoing work with DF, and perhaps measuring IL-6 would have been more informative from a collective examination of past animal models [37]. Regardless, models of inflammation have long demonstrated that a collective series of cytokines can potentially be increased during inflammation, but not necessarily multiple ones for any single model [38]. It is for this reason that two separate studies seemed more logical to determine further mechanistic insights into past and future clinical studies. Yet, the complexities of the immune system and the ability of numerous compounds to have bidirectional roles in specific disease activity are daunting and will always represent a limitation with this kind of basic science investigation [39, 40]. Regardless, the sheer number of clinical studies and *in vitro* mechanism of action work on DF at this point appears, in our opinion, to have hopefully established a positive paradigm in the field of immune modulating dietary supplements.

## 5. Conclusions

The combined positive results of studies 1 and 2 suggest that DF may act by modulating multiple acute and potentially chronic immunologic and inflammatory control mechanisms. These findings add to the clinical observations in the five previous randomized trials and perhaps suggest efficacy in other areas of immune support. Further clinical and laboratory research should be conducted to better understand the areas where DF may continue to provide beneficial clinical results.
